# Supplementary material for: SyNDI: synchronous network data integration framework
Source: BMC Bioinformatics. 2018 Nov 6;19:403. doi: 10.1186/s12859-018-2426-5 (PMC6219086; doi:10.1186/s12859-018-2426-5)
Supplement: Supplementary file 5 — Files of the example presented in Additional file 4. This additional file is a zip package that contains all files used in Additional file 4. Detailed descriptions of each of these files are presented in a README file included in this zip package. (GZ 30411 kb) [file 12859_2018_2426_MOESM5_ESM.gz › SyNDITools/Meme2Fimo/meme_4.9.0_4.tar.gz!/meme_4.9.0/doc/dreme-tutorial.html]

DREME Tutorial


# DREME Tutorial

## Overview

DREME is a tool for discovering short regular expression motifs which
are enriched in the provided dataset. It is limited to working in DNA
as the large combination space for amino acids makes DREME's approach
unfeasable. DREME also has the capability to use two datasets to
find motifs which are enriched in one when compared to the other.

## How DREME works

Note the following referrs to the sequence set in which you are finding
motifs in as the positive sequences and to the comparative sequence
set as the negative sequences.

1. If there are no negative sequences provided then di-nucleotide
   shuffle the postive sequences to create one.
2. Count the number of positive sequences and the number of negative
   sequences.
3. Find all unique subsequences with no ambiguity characters which
   have a length in the range given (3 to 8 nucleotides by default)
   in the positive sequences.
4. For each of the subsequences
   1. Count the number of sequences it occurs in for the positive and
      negative sequences.
   2. Use Fisher's exact test to determine the significance.
   3. Add the subsequence to a sorted (by p-value) set of regular
      expression motifs.
5. Repeatedly pick the top motifs (default 100) to generalise
   by replacing one position with each possible ambiguity code and
   estimating the resultant p-value. This is done enough times to allow
   each position to have an ambiguity code.
6. For each of the top (default 100) generalized RE motifs
   1. Count the number of sequences matched in the positive and negative
      sequences.
   2. Use Fisher's exact test to determine the significance.
7. Pick the best RE motif and (assuming it meets the E-value threshold)
   scan for all matching sites to build up a frequency matrix and report
   it.
8. Mask the matched sites with the wildcard character 'N'.
9. If the limits have not been met then loop back to step 3 to find
   more motifs.

## Sequence set

DREME works best with lots of short (~ 100bp) sequences. If you have a
couple of long sequences then it might be benificial to split them into
many smaller (~ 100bp) sequences. With ChIP-seq data we recommend using
100bp regions around the peaks.

## Comparative sequence set

DREME always uses a comparative sequence set but you don't have to supply
it as DREME can create it by using di-nucleotide shuffling. If you wish
to use your own sequence set then there are a few guidlines you should
follow.

The sequence lengths of the comparative sequences should be roughly
the same as the sequences to search for motifs. This is because the null
model assumes that the probability of finding a match in a sequence in
either sequence set will be roughly the same for an uninteresing motif.
If the comparative sequences are longer this provides more
locations that the motif could match making it more likely it will match
and hence skewing the p-value calculations possibly excluding a motif
you would be interested in.
